# Correlation between atherogenic index of plasma and coronary artery disease in males of different ages: a retrospective study

**DOI:** 10.1186/s12872-022-02877-2

**Published:** 2022-10-09

**Authors:** Lei Hong, Yu Han, Chunfeng Deng, Aihua Chen

**Affiliations:** 1 Department of Cardiology, Heart Center, Zhujiang Hospital, Southern Medical University, NO. 253, Gongye Avenue, Guangzhou, 510282 China; 2 Guangdong Provincial Biomedical Engineering Technology Research Center for Cardiovascular Disease, NO. 253, Gongye Avenue, Guangzhou, 510282 China; 3 Laboratory of Heart Center, Zhujiang Hospital, Southern Medical University, NO. 253, Gongye Avenue, Guangzhou, 510282 China; 4 Guangdong Provincial Key Laboratory of Shock and Microcirculation, NO. 253, Gongye Avenue, Guangzhou, 510282 China; 5 Department of Cardiology, Longgang Central Hospital, Shenzhen, 518116 China; 6 Centre for Human and Applied Physiological Sciences, Faculty of Life Sciences and Medicine, King’s College London, London, SE1 1UL England, UK; 7 Department of Nephrology, Longgang Central Hospital, Shenzhen, 518116 China

**Keywords:** Coronary artery disease, Atherogenic index of plasma, Age

## Abstract

**Background:**

Atherogenic index of plasma (AIP) as a newly discovered blood lipid parameter was shown to be strongly correlated with coronary artery disease (CAD). However, the blood lipid levels changed with age, so we speculated that the correlation between AIP and CAD was also affected by age.

**Methods:**

A retrospective study was performed on male patients with confirmed or suspected CAD who underwent coronary angiography (CAG) from July 2017 to March 2021. Patients were divided into younger, middle early, middle late, and elderly groups. Patients in each age group were further divided into the non-coronary artery disease (n-CAD) and CAD subgroups according to the CAG diagnostic results. The relationship between blood lipid parameters and CAD was assessed in each age group.

**Results:**

Age-dependent changes of blood lipid levels were mainly found in CAD patients but not in n-CAD patients. With increased age, the levels of triglyceride, total cholesterol, low-density lipoprotein cholesterol (LDL-C) and AIP were gradually decreased, whereas high-density lipoprotein cholesterol concentration was increased in CAD patients. Multivariate logistic regression analyses showed that AIP was an independent risk factor for CAD in middle early (OR 2.601; CI 1.160–5.832, *P* = 0.02) and middle late age group (OR 2.707, CI 1.201–6.100, *P* = 0.016), but not in the younger and elderly groups. LDL-C was an independent risk factor for CAD in all age groups. The areas under curve of AIP for detecting CAD in the middle early and middle late age groups were not higher than that of other blood lipid parameters.

**Conclusion:**

Although this was a single-center study for males only, the correlation between AIP level and CAD risk was age-dependent. AIP was an independent risk factor for CAD in the middle-aged groups. However, the predictive value of AIP for detecting CAD was not better than that of the traditional blood lipid parameters such as LDL-C.

## Background

Cardiovascular disease is the leading cause of death in China [1]. Dyslipidemia, especially elevated low-density lipoprotein cholesterol (LDL-C), is one of the major risk factors for cardiovascular disease. Thus, LDL-C has always been the most important target in the treatment of patients with coronary artery disease (CAD) [2].

Previous studies have shown [3] that for every 1 mmol/L reduction in LDL-C, coronary events can be decreased by 23%. Even if LDL-C concentration is maintained at 1.99 mmol/L by intensive therapy with high doses of statin drugs, the incidence of cardiovascular events is lowered from 10.9 to 8.7% [4]. This means that despite implementing standard lipid-lowering therapy and controlling risk factors such as blood pressure and glycaemia, patients may still develop cardiovascular events, which is also known as residual cardiovascular risk [5]. Hence, applying LDL-C to assess the risk of CAD still faces limitations. It is necessary to look for new blood lipid parameter to better predict cardiovascular diseases.

In recent years, some new blood lipid parameters have been proposed. These new parameters have evolved from traditional blood lipid parameters such as triglyceride (TG), total cholesterol (TC), high-density lipoprotein cholesterol (HDL-C), and LDL-C, including non-HDL-C, LDL-C/HDL-C, non-HDL-C/HDL-C, TC/HDL-C, and the atherogenic index of plasma (AIP). AIP, a logarithmically transformed ratio of TG/HDL-C, is considered a marker of Arteriosclerosis [6]. Studies [5] have shown that patients with residual cardiovascular risk were mainly characterized by having high TG and low HDL-C levels. AIP can directly reflect TG and HDL-C, therefore, more and more research has been carried out on the relationship between AIP and CAD risk [6–8]. The results suggest that AIP is an independent predictor for CAD, with a higher predictive power compared with traditional blood lipid parameters. On the contrary, some studies have shown that AIP seems not to be significantly correlated with the occurrence and development of CAD in specific populations [9–11]. The inclusion criteria, enrollment, gender ratio, race, eating habits, and the statistical methods were different in these studies. As a result, findings were inconsistent. Therefore, we believe that if the patients were stratified by age with the same inclusion criteria and statistical methods to observe the correlation between AIP and CAD in different age groups, the results will be more reliable.

A number of epidemiological studies have shown that blood lipid levels change with age, though the influence of age on blood lipid level may vary [12–15]. For example, TG or LDL-C levels are decreased in the elderly, while HDL-C level was increased in this population [12]. Another survey on patients with myocardial infarction also showed that the TG and TC levels in elderly patients were lower than those in young and middle-aged patients [15]. Therefore, we believed that age must be particularly considered when discussing the correlation between lipid parameters and cardiovascular diseases. However, at present, there are few relevant studies in this area. As expected, all these studies showed significant differences in blood lipids between genders, Therefore, the aim of this study was to investigate the relationship between AIP and CAD in male patients in different age groups.

## Methods

### Population and study design

**Fig. 1:**
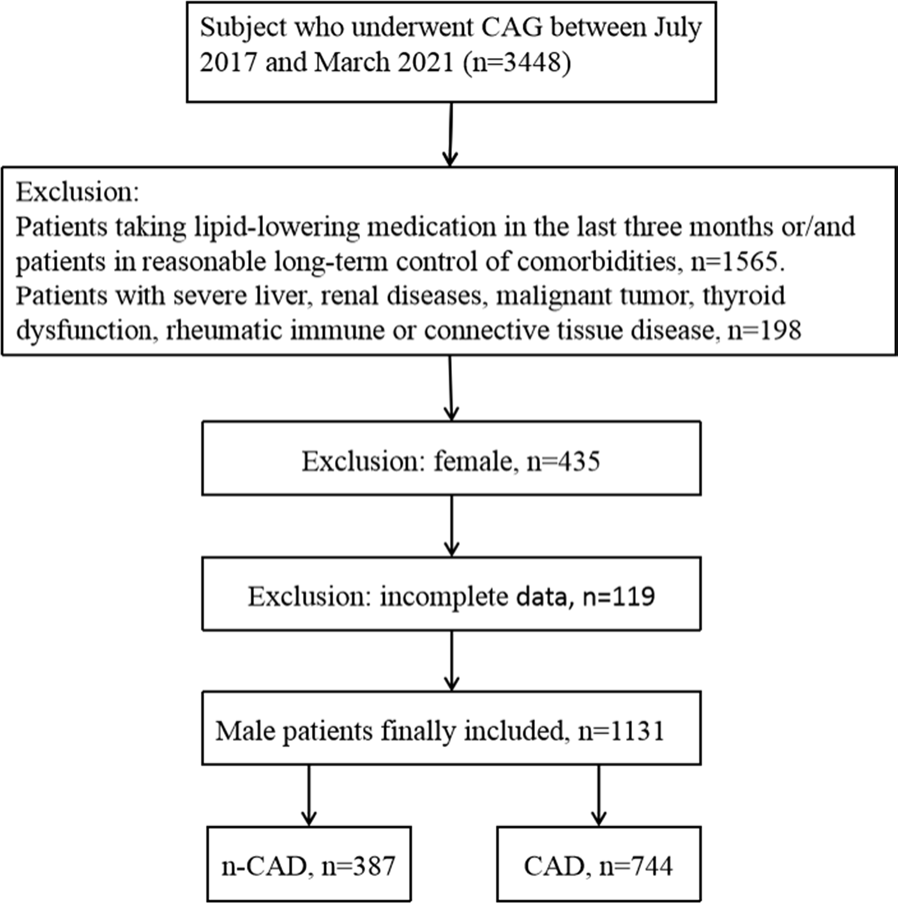
The flow chart of the study

Patients with confirmed or suspected CAD who were admitted to the Department of Cardiology of Longgang Central Hospital and underwent coronary artery angiography (CAG) from July 2017 to March 2021 were enrolled. Patients’ data were retrospectively collected from the electronic medical record system of the hospital. The inclusion criteria were: (1) patients with elevated troponin (CTN), typical abnormal ECG, and typical clinical manifestations of CAD who were diagnosed with CAD (unstable angina, non st-elevation myocardial infarction, or st-elevation myocardial infarction); and (2) patients with atypical clinical manifestations who underwent CAG to identify coronary artery lesions. The exclusion conditions were: (1) female patients; (2) patients taking lipid-lowering medication in the last three months; (3) patients with reasonably controlled essential hypertension (EH), Diabetes mellitus (DM), hyperuricemia (HUA), and other comorbidities; (4) patients with severe liver and renal diseases, malignant tumor, thyroid dysfunction, and rheumatic immune or connective tissue diseases; and (5) those with incomplete data. Finally, 1131 male patients were enrolled. The flow chart of patient enrolment process is shown in Fig. 1.

All patients were divided into four age groups [16]: younger (≤ 34 years), middle early (35–49 years), middle late (50–64 years), and elderly (≥ 65 years) groups. Then patients in each age group were further divided into non-coronary artery disease (n-CAD) and CAD subgroups based on CAG results. The relationship between serum lipid parameters and CAD was assessed in n-CAD and CAD groups.

### Case collection and definition

Patients’ data including age, smoking history, EH, DM, and HUA complications, and CAG results were collected from the electronic medical record system of Longgang Central Hospital. Venous blood was collected from all patients within 12 h after admission, and the levels of blood lipids including TG, TC, LDL-C and HDL-C were measured. AIP was calculated as log_10_ (TG/HDL-C).

CAD was diagnosed according to the 1979 WHO criteria [17]. n-CAD was defined as the stenosis of a major coronary artery stenosis of no more than 50%. EH was defined as systolic blood pressure ≥ 140 mmHg and/or diastolic blood pressure ≥ 90 mmHg on multiple measurements, or with a prior history of EH. DM was defined as fasting glucose ≥ 7.0 mmol/L and/or 2-h glucose ≥ 11.1 mmol/l, or with a previous history of DM. HUA was defined as fasting serum uric acid ≥ 425umol/L or with a previous history of HUA. Smokers were defined as those who smoked at least one cigarette a day for more than one year.

### Statistical analysis

Statistical analysis was performed using SPSS 22.0 (IBM Corp, Armonk, NY) and GraphPad Prism 5.01 (GraphPad Software Inc. La Jolla, CA, USA). Shapiro–Wilk test was used to check if a continuous variable followed a normal distribution. Data with a normal distribution were represented by mean ± standard deviation (SD), and t-test or ANOVAs was used. Data with a skewed distribution were represented by median (25th-75th), and Mann–Whitney U or Kruskal–Wallis was used. Multiple comparisons for blood lipid parameters among age groups were carried out by the ANOVA with post hoc Dunnet T3 test. Categorical variables were expressed as frequencies and percentages, and Chi-square test was used. Binomial univariate logistic regression analyses were conducted to assess the relationship between blood lipid parameters and CAD in different age groups. Multivariate logistic regression analysis with adjustment of covariates including smoking, EH, DM, and HUA was further performed. Hosmer–Lemeshow and collinearity tests were used to evaluate the predictive probability of each lipid parameter for CAD risk in the multivariate model. Then a receiver operating characteristic curve (ROC) curve and areas under curve (AUC) were drawn to analyze the auxiliary value of lipid parameters for CAD. Two-sided *P* < 0.05 was considered statistically significant.

## Results

The general characteristics of the patients are shown in Table 1. The age of the enrolled subjects ranged from 19 to 92 years, with 145 cases in the younger group (12.82%), 396 cases in the middle early group (35.01%), 395 cases in the middle late group (34.92%), and 195 cases in the elderly group (17.24%). The serum lipid levels of different age groups were significantly different, and the prevalence of CAD, EH and DM comorbidities increased with age.

**Table 1:** The baseline data of the enrolled patients

	Younger (19–34y)	Middle early (35–49y)	Middle late (50–64y)	Elderly (65y–92)	*P* value
N (%)	145 (12.82%)	396 (35.01%)	395 (34.92%)	195 (17.24%)	NA
Age	32 (28, 34)	44 (41, 47)	55 (52, 60)	71 (67, 75)	NA
CAD (%)	60 (41.38)	253 (63.89)	288 (72.91)	143 (73.33)	0.000
EH (%)	29 (20)	159 (40.15)	187 (47.34)	108 (55.38)	0.000
DM (%)	12 (8.28)	59 (14.9)	78 (19.75)	44 (22.56)	0.001
HUA (%)	65 (44.86)	142 (35.86)	123 (31.14)	73 (37.44)	0.117
Tobacco (%)	78 (53.79)	276 (69.7)	266 (67.34)	104 (53.33)	0.000
TG (mmol/L)	1.54 (0.965, 2.65)	1.61 (1.11, 2.35)	1.45 (1.02, 2.05)	1.13 (0.9, 1.56)	0.000
TC (mmol/L)	4.44 (3.74, 5.13)	4.34 (3.74, 5.05)	4.41 (3.79, 5.08)	4.08 (3.53, 4.66)	0.001
HDL-C (mmol/L)	0.97 (0.81, 1.165)	0.93 (0.79, 1.09)	0.93 (0.81, 1.1)	1.00 (0.82, 1.22)	0.011
LDL-C (mmol/L)	3.06 (2.45, 3.69)	2.99 (2.36, 3.60)	3.04 (2.49, 3.65)	2.81 (2.20, 3.36)	0.009
AIP	0.217 ± 0.369	0.246 ± 0.297	0.200 ± 0.301	0.090 ± 0.260	0.000

Kruskal–Wallis Test were used for TG, TC, HDL-C, LDL-C; χ2 test were used for distributions of CAD, EH, DM, HUA, and tobacco, ANOVAs were used for AIP. NA: statistical test was not performed

*CAD* Coronary artery disease, *EH* essential hypertension, *DM* Diabetes mellitus, *HUA* Hyperuricemia, *TG* Triglyceride, *TC* Total cholesterol, *HDL-C* High density lipoprotein cholesterol, *LDL-C* Low density lipoprotein cholesterol, *AIP* Atherogenic index of plasma

**Fig. 2:**
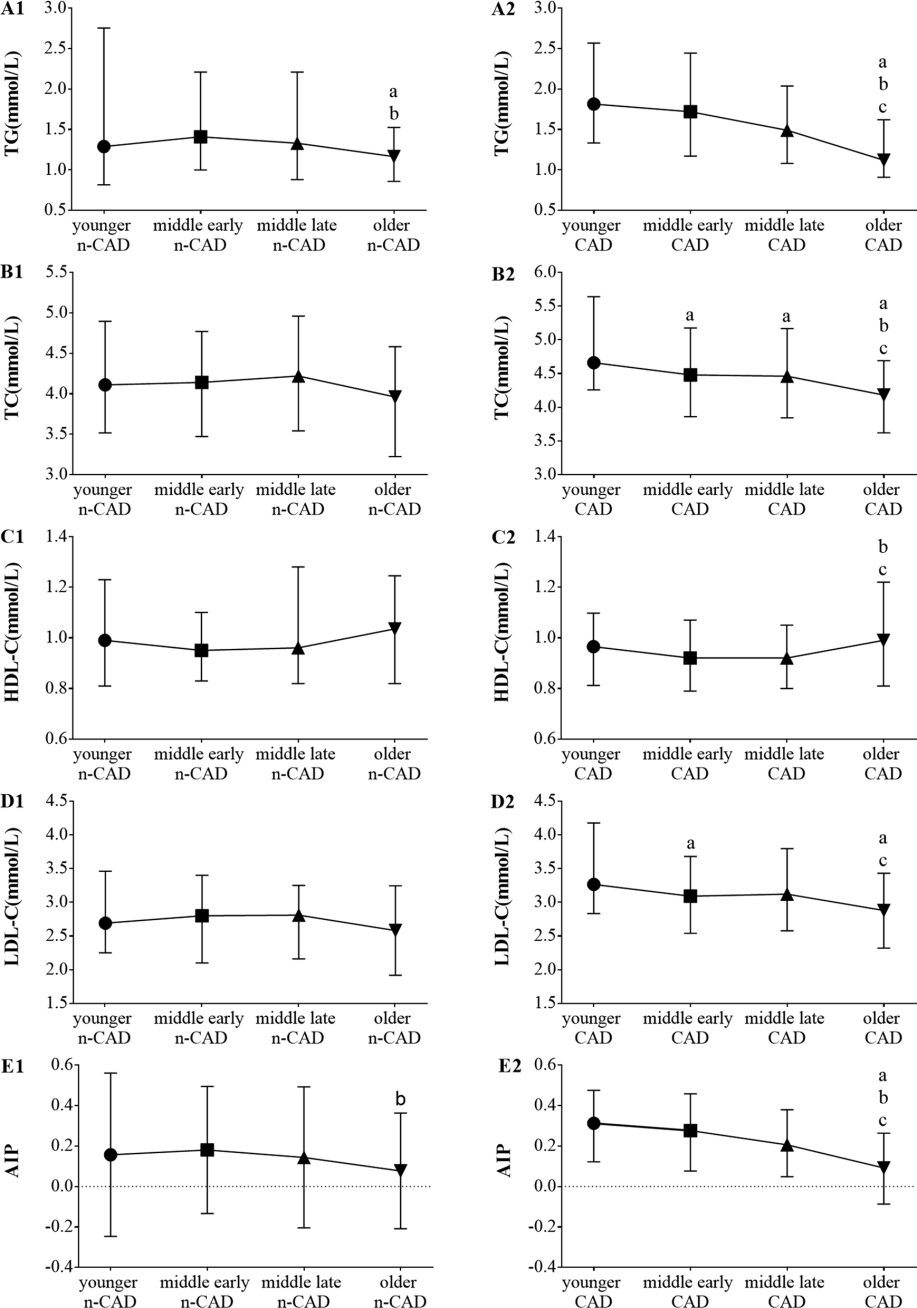
Blood lipid indicator are represented by median with interquartile range. post hoc Dunnet T3 test was used. **a** significant difference at *P* < 0.05 compared with the younger group; **b** significant difference at *P* < 0.05 compared with the middle early group; **c** significant difference at *P* < 0.05 compared with the middle late group. *CAD* Coronary artery disease, *n-CAD* non-Coronary artery disease, *TG* Triglyceride, *TC* Total cholesterol, *HDL-C* High density lipoprotein cholesterol, *LDL-C* low density lipoprotein cholesterol, *AIP* atherogenic index of plasma

As shown in Fig. 2, for n-CAD patients, the levels of TG, and AIP in the older group were lower. The blood lipid levels of the CAD group showed a more obvious age-dependent trend. With increased age, the levels of TG, TC, LDL-C and AIP were decreased gradually, and HDL-C level was the highest in the older group.

**Fig. 3:**
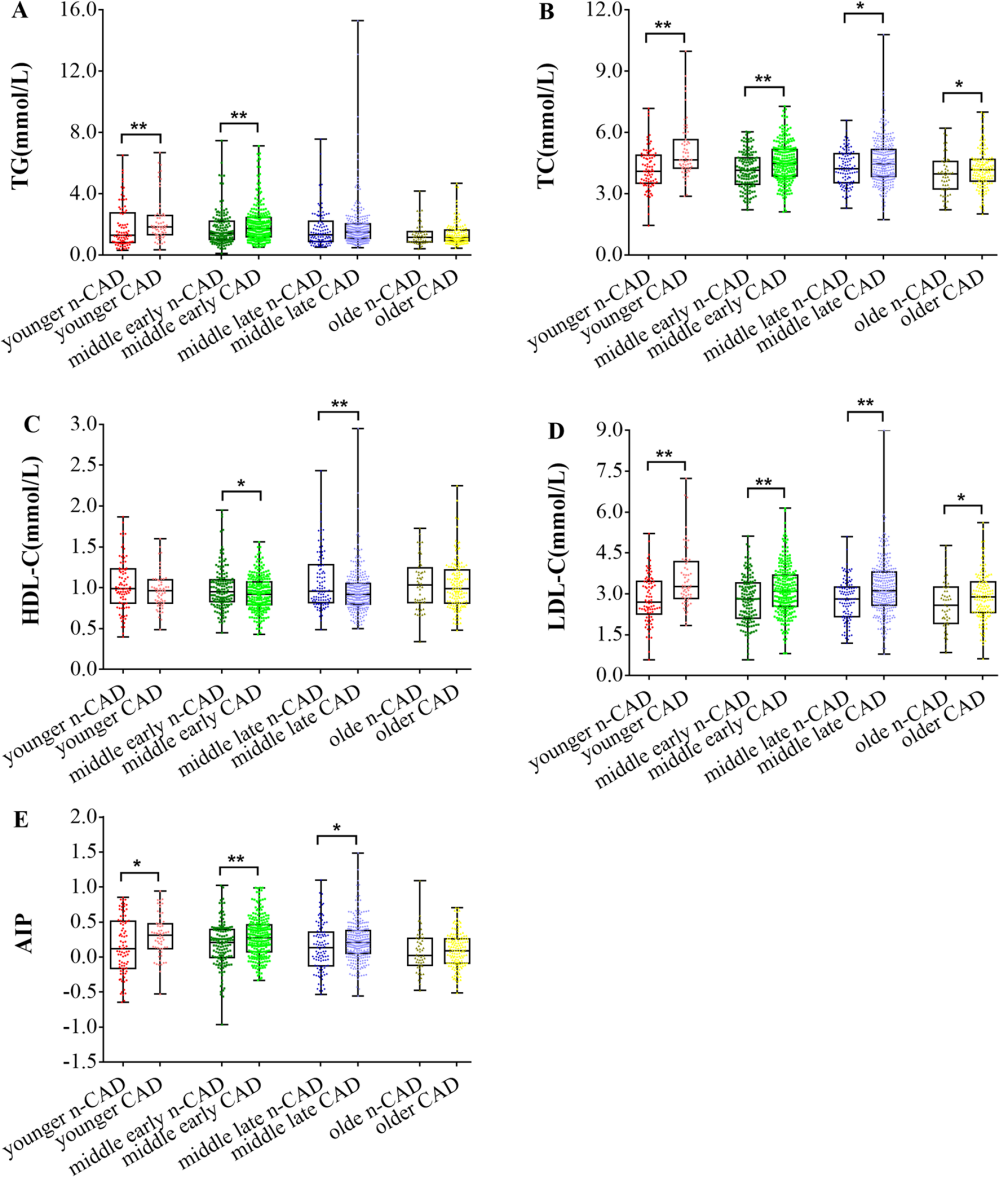
The distributions of blood lipids in each age group are illustrated by the Box and Whisker Plot, and the dotted lines represent the values ranging from minimum to maximum. T-test was used when both groups had normally distributed data; otherwise, Mann–Whitney U test was used. * *P* < 0.05, ** *P* < 0.01. *CAD* Coronary artery disease, *n-CAD* non Coronary artery disease, *TG* Triglyceride, *TC* Total cholesterol, *HDL-C* High density lipoprotein cholesterol, *LDL-C* low density lipoprotein cholesterol, *AIP* atherogenic index of plasma

As shown in Fig. 3, TG level in younger and middle early-aged patients with CAD was higher than that in patients of similar age without CAD. HDL-C concentration in middle-aged patients with CAD was lower than that in patients of similar age without CAD. Younger and middle-aged patients had higher AIP level when they suffered from CAD, whereas CAD and n-CAD elderly patients showed no difference in AIP level. Moreover, CAD patients had higher TC and LDL-C levels compared with n-CAD patients, regardless of age.

Lipid parameters were firstly included in univariate logistic regression analysis ( Table 2), and those with a *P* < 0.05 were subsequently entered into the multivariate logistic regression analysis. Risk factors of CAD such as smoking, EH, DM and HUA were adjusted in the multivariate model. Hosmer–lemeshow test confirmed good fitting of each model, and collinearity test excluded non-obvious collinearity among factors. The results showed that TC was an independent risk factor for CAD in younger, middle early and middle late groups (*P* < 0.01), whereas AIP was an independent risk factor for CAD in middle early and middle late groups (*P* < 0.05). Only LDL-C was an independent risk factor for CAD in all age groups (*P* < 0.05) (Table 3).

**Table 2:** Univariate logistic regression analysis of the relationship between blood lipid parameters and CAD

Variables	Younger (19–34y)		Middle early (35–49y)		Middle late (50–64y)		Elderly (65y–92)	
	OR (95% CI)	*P*	OR (95% CI)	*P*	OR (95% CI)	*P*	OR (95% CI)	*P*
TG	1.225 (0.973–1.544)	0.085	1.235 (1.019–1.496)	0.031	1.040 (0.882–1.229)	0.633	1.060 (0.678–1.6570)	0.797
TC	1.998 (1.405–2.841)	0	1.543 (1.227–1.94)	0	1.367 (1.075–1.738)	0.011	1.409 (0.990–2.006)	0.057
HDL-C	0.405 (0.119–1.375)	0.147	0.288 (0.118–0.707)	0.007	0.248 (0.115–0.537)	0.000	0.847 (0.298–2.408)	0.755
LDL-C	2.266 (1.503–3.416)	0	1.580 (1.238–2.016)	0	1.738 (1.325–2.278)	0.000	1.499 (1.026–2.189)	0.036
AIP	3.056 (1.186–7.874)	0.021	3.335 (1.612–6.899)	0.001	2.456 (1.131–5.333)	0.023	1.304 (0.380–4.467)	0.673

*TG* Triglyceride, *TC* Total cholesterol, *HDL-C* High density lipoprotein cholesterol, *LDL-C* Low density lipoprotein cholesterol, *AIP* Atherogenic index of plasma, *OR* Odds ratio, *CI* Confidence interval

**Table 3:** Multivariate logistic regression analysis of the relationship between blood lipid parameters and CAD

Variables	Younger (19-34y)		Middle early (35-49y)		Middle late (50-64y)		Elderly (65y-92)	
	OR (95% CI)	*P*	OR (95% CI)	*P*	OR (95% CI)	*P*	OR (95% CI)	*P*
TG	/	/	1.168 (0.949–1.437)	0.142	/	/	/	/
TC	1.847 (1.288–2.647)	0.001	1.687 (1.317–2.162)	0	1.522 (1.183–1.959)	0.001	/	/
HDL-C	/	/	0.419 (0.162–1.087)	0.074	0.257 (0.116–0.570)	0.001	/	/
LDL-C	2.182 (1.408–3.380)	0	1.774 (1.362–2.311)	0	1.928 (1.452–2.560)	0.000	1.539 (1.046–2.264)	0.029
AIP	1.753 (0.619–4.964)	0.29	2.601 (1.160–5.832)	0.02	2.707 (1.201–6.100)	0.016	/	/

*TG* Triglyceride, *TC* Total cholesterol, *HDL-C* High density lipoprotein cholesterol, *LDL-C* Low density lipoprotein cholesterol, *AIP* Atherogenic index of plasma, *OR* Odds ratio, *CI* Confidence interval

**Fig. 4:**
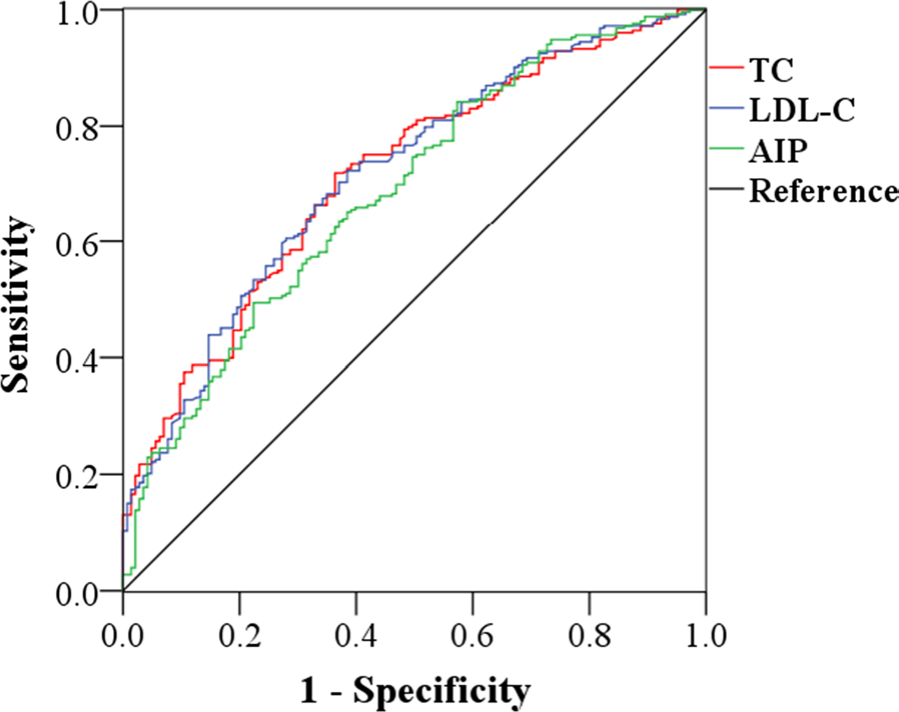
ROC-AUC analysis of TC, LDL-C and AIP in the middle early group. *TC* Triglyceride, *LDL-C* low density lipoprotein cholesterol, *AIP* atherogenic index of plasma

**Fig. 5:**
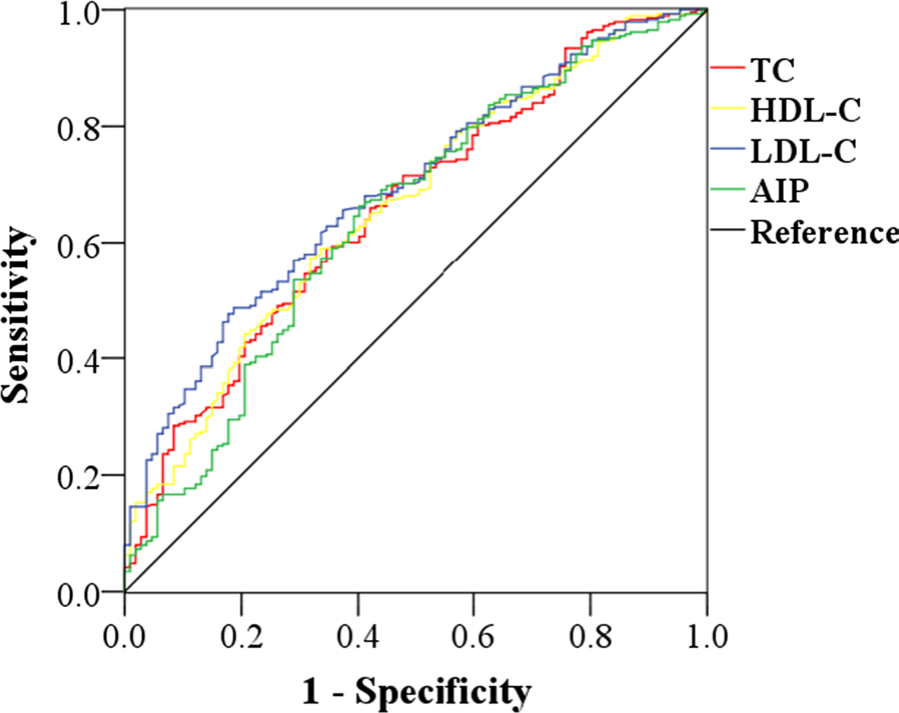
ROC-AUC analysis of TC, HDL-C, LDL-C and AIP in the middle late group. *TC* Triglyceride, *HDL-C* High density lipoprotein cholesterol, *LDL-C* low density lipoprotein cholesterol, *AIP* atherogenic index of plasma

Multivariate logistic regression analysis showed that AIP was an independent risk factor for CAD in the middle early and middle late groups. Therefore, we performed ROC analysis and used AUC to further evaluate and compare the predictive ability of traditional blood lipid parameters and AIP for CAD in these two age groups, as shown in Table 4 and Fig. 4. The results suggested that the AUCs of TC, LDL-C, and AIP were relatively close and all had a certain predictive value for CAD. The diagnostic values of TC, HDL-C, LDL-C, and AIP for CAD in the middle late group were shown in Table 4 and Fig. 5. The results indicated that the above blood lipid parameters had similar predictive ability for CAD. However, the AUC of AIP in these two middle age groups was slightly lower than those of traditional blood lipid parameters.

**Table 4:** Comparisons of AUC values of different blood lipid parameters

variables	Middle early (35–49y)		Middle late (50–64y)	
	AUC (95%CI)	*P*	AUC (95%CI)	*P*
TC	0.715 (0.664–0.766)	0.000	0.661 (0.601–0.720)	0.030
HDL-C	–	–	0.662 (0.603–0.722)	0.030
LDL-C	0.716 (0.665–0.768)	0.000	0.691 (0.635–0.747)	0.029
AIP	0.687 (0.633–0.741)	0.000	0.645 (0.582–0.707)	0.032

*TC* Triglyceride, *HDL-C* High density lipoprotein cholesterol, *LDL-C* Low density lipoprotein cholesterol, *AIP* Atherogenic index of plasma, *AUC* Areas under curve, *CI* Confidence interval

## Discussion

This study was focused on male patients, because there were significant difference in lipid metabolism between male and female, and the predominance of male participants might interfere with the interpretation of the results. The number of premenopausal females who underwent CAG examination was significantly less than that of males of the same age, which might affect the authenticity of the statistical results as the menstrual cycle could also affect the blood lipid levels and the incidence of CAD in females. Therefore, we believed that gender preference was necessary.

This study demonstrated that TG, TC, LDL-C, and AIP levels exhibited an obvious downward trend with aging in CAD patients, but this trend was not obvious in patients without CAD. TG, TC, and LDL-C levels were shown to be positively correlated with CAD, whereas HDL-C level was negatively correlated with CAD. According to the results above, it seemed that the blood lipids in elderly patients were maintained at a healthier level. However, as shown in Table 1, the elderly age group had the highest incidence of CAD, which might be due to the following reasons. Firstly, non-cardiogenic chest pain misdiagnosed as CAD is more common in young people compared with the elderly, so the actual incidence of CAD in young people is relatively lower [18]. Secondly, dyslipidemia is only one cause of CAD in the older people. The elderly have a high chance to develop EH and DM comorbidities and other diseases with a long course, and are more susceptible to coronary artery involvement. Furthermore, some scholars [19] have pointed out that although the severity of dyslipidemia in the elderly is weaker than that in the young and middle-aged people, age itself is an important risk factor for CAD, and the compensatory ability and target organ function of older adults were reduced. Therefore, dyslipidemia in the elderly population, especially high LDL-C level, is more pathogenic.

In this study, the changing trend of blood lipid levels with aging was not obvious in n-CAD population, which might be attributed to better blood lipid metabolism and healthier eating habits of the n-CAD population.

Tobacco is reported to be an independent risk factor for CAD. We found that the proportion of smokers in the elderly group, which had the highest rate of CAD, was relatively low. We speculated that this result might be related to the fact that many long-term smokers were admitted to the Department of respiratory or oncology and thus were not enrolled in our study. In addition, many elderly smoking patients had hypertension, diabetes, and cardiovascular diseases, they usually took statins in previous treatments and were excluded in this trial.

There are numerous studies on AIP. Dobiasova M et al. first explored the significance of AIP and arteriosclerosis. They found that AIP was significantly negatively correlated with lipoprotein particle size and suggested that AIP could be a predictor of CAD [6, 20]. A case control study of 696 postmenopausal women conducted by Wu TT et al. showed that after adjusting for multiple clinical factors, AIP could be an independent risk factor for CAD [7]. Guelker JE et al. analyzed the correlation between artery total occlusion and AIP in 317 patients, the majority of patients were male (82.6%), and mean age was 61 years. The results showed that increased AIP was associated with longer occlusion length, stent routes and a higher number of implanted stents [8]. Zhan YQ et al. conducted a retrospective study on acute coronal syndrome (ACS) in 376 patients, with the average age of approximately 60 years. The results showed that AIP was higher in the ACS group compared to the control group [21]. Yunke Z et al. [22] conducted a 3-year study to evaluate the TG/HDL-C association with CAD and found that the TG/HDL-C ratio could also predict new onset of heart failure in CAD patients. The association of AIP with cerebrovascular disease has also been explored. Wang C et al. conducted a large-scale survey involving 11,495 people above 35 years. Logistic regression analysis showed that irrespective of gender, AIP was independently associated with the occurrence of ischemic stroke [23]. A meta-analysis on the correlation between diabetes mellitus and blood lipids showed that compared with other blood lipid indexes (TG, TC, HDL-C, LDL-C), AIP may be more closely associated with the risk of diabetes mellitus [24]. However, the true clinical value of AIP was still controversial.

Studies on very young CAD Chinese patients [25] and Mexican patients aged 18–22 years [26] showed that AIP level was significantly correlated with CAD or other CAD risk factors. However, In the present study, AIP was not an independent risk factor for CAD in the younger age group. Previous studies have shown that AIP can indirectly reflect the diameter of sd-LDL particles which is closely related to arteriosclerosis. However, Goliasch et al. found that there was no correlation between sd-LDL particles and early onset CAD in people aged 40 years and below [27]. Some studies also mentioned that arterial endothelial dysfunction caused by smoking or genetic factors might play an important role in the development of atherosclerosis in young people [28]. Based on clinical experience, we also believed that staying up late, smoking, and excessive pressure might be more likely associated with CAD risk in young people compared with abnormal blood lipid levels.

The relationship between AIP and CAD in elderly patients was more controversial. Studies have shown that AIP was strongly correlated with all-cause mortality of the elderly aged 65–85 years [29]. However, a study on the elderly population over 60 years old showed that the correlation between AIP and all-cause mortality only appeared in females but not in males [11]. A study on menopausal females in China also indicated that AIP was an ideal predictor for CAD in this population [7]. Interestingly, another on Chinese people over 65 years showed the opposite result that AIP was an independent risk factor for CAD in elderly males [30]. In order to avoid negative AIP values, they calculated AIP as log_10_(TG/HDL-C*10), rather than log_10_(TG/HDL-C). It was not known if the two measurement methods could lead to different results. AIP was not seen as an independent risk factor for elderly CAD male patients in this study. Since we derived AIP from the TG to LDL-C ratio, and TG level was the lowest and HDL-C level was the highest in the elderly group, which inevitably resulted in the lowest AIP value. However, the proportion of CAD patients was the highest in the elderly group, which might explain non-correlation between AIP and CAD in older patients.

In this study, AIP was demonstrated to be an independent risk factor for CAD patients aged 35–64 years. However, the AUC result did not support some previous conclusions that AIP was superior to traditional blood lipid parameters such as LDL-C in terms of CAD detection, so we believed that AIP was not an ideal predictive biomarker of CAD. CAD diagnosis and treatment guidelines have recommended that LDL-C is the most important target for lipid treatment, whereas the role of AIP was not mentioned. Although many studies have shown a relationship between AIP and cardiovascular diseases, more authoritative conclusions were still lacking.

### Limitation

There were some limitations in this study. This was a single-center study focused on male CAD patients. Since this was a retrospective study, we were also unable to obtain more information about the patients, such as lifestyle/eating habits, personality, work stress, financial status, etc. In addition, many other similar studies included indicators such as heart rate, blood pressure, blood sugar, blood routine, and inflammatory factors were excluded in this study as the fluctuation under the stress of onset of the disease and admission. At the same time, many patients in China would use lipid-lowering drugs for secondary prevention of CAD, these preventive measures would reduce blood lipids and delay the progression of atherosclerosis, so these patients were also excluded.

## Conclusion

Blood lipid levels in CAD patients changed significantly with age. The correlation of AIP with CAD was mainly implicated in the middle-aged population aged between 35 and 64 years old, and the predictive value of AIP for CAD was not found to be superior to the traditional lipid parameter. Therefore, cardiologists should consider the age of patients when using the AIP to predict the risk of CAD.

## Data Availability

The datasets used and/or analyzed during the present study are available from the corresponding author on reasonable request (E-mail: zj_chenaihua@126.com).

## Abbreviations

AIP: Atherogenic index of plasma
CAD: Coronary artery disease
n-CAD: Non-coronary artery disease
EH: Essential hypertension
DM: Diabetes mellitus
HUA: Hyperuricemia
TG: Total cholesterol
TC: Triglyceride
HDL-C: High density lipoprotein cholesterol
LDL-C: Low density lipoprotein cholesterol
AUC: Area under the curve
OR: Odds ratio
ROC: Receiver operating characteristic
CI: Confidence interval
WHO: World Health Organization
